# Improving mental health through integration with primary care in rural Karnataka: study protocol of a cluster randomized control trial

**DOI:** 10.1186/s12875-018-0845-z

**Published:** 2018-09-11

**Authors:** Krishnamachari Srinivasan, Amanda Mazur, Prem K. Mony, Mary Whooley, Maria L. Ekstrand

**Affiliations:** 1 Division of Mental Health and Neurosciences, St. John’s Research Institute, St. John’s National Academy of Health Sciences, Bangalore, Karnataka India; 2 Department of Psychiatry, St John’s Medical College Hospital, Bangalore, Karnataka India; 3 Division of Prevention Sciences, University of California, San Francisco, USA; 4 Division of Epidemiology and Community Health, St. John’s Medical College and Research Institute, St. John’s National Academy of Health Sciences, Bangalore, India; 5 Division of Cardiology, University of California, San Francisco, USA; 6 San Francisco Veterans Affairs Medical Center, San Francisco, CA USA

**Keywords:** Mental health, Chronic disease, Collaborative care, Randomized controlled trial, India

## Abstract

**Background:**

People who are diagnosed with both mental and chronic medical illness present unique challenges for the health care system. In resource-limited settings, such as rural India, people with depression and anxiety are often under-served, due to both stigma and lack of trained providers and resources. These challenges can lead to complications in the management of chronic disease as well as increased suffering for patients, families and communities. In this study, we evaluate the effects of integrating mental health and chronic disease treatment of patients in primary health care (PHC) settings using a collaborative care model to improve the screening, diagnosis and treatment of depression in rural India.

**Methods:**

This study is a multi-level randomized controlled trial among patients with depression or anxiety and co-morbid diabetes, or cardiovascular disease. Aim 1 examines whether patients screened at community health-fairs are more likely to be diagnosed and treated for these co-morbid conditions than patients screened after presenting at PHCs. Aim 2 evaluates the impact of collaborative care compared to usual care in a cluster RCT, randomizing at the level of the PHCs. Intervention arm PHC staff are trained in mental health diagnoses, treatment, and the collaborative care model. The intervention also involves community-based “Healthy Living groups” co-led by Ashas, using cognitive-behavioral strategies to promote healthy behaviors. The primary outcome is severity of common mental disorders, with secondary outcomes being diabetes and cardiovascular risk, staff knowledge and patient perceptions.

**Discussion:**

If effective, our results will contribute to the field in five ways: 1) expand on implementation research in low resource settings by examining how multiple chronic diseases can be treated using integrated low-cost, evidence-based strategies, 2) build the capacity of PHC staff to diagnose and treat mental illness within their existing clinic structure and strengthen referral linkages; 3) link community members to primary care through community-based health fairs and healthy living groups; 4) increase mental health awareness in the community and reduce mental health stigma; 5) demonstrate the potential for intervention scale-up and sustainability.

**Trial registration:**

http://Clinicaltrials.gov: NCT02310932 registered December 8, 2014 URL: https://clinicaltrials.gov/ct2/show/record/NCT02310932; Clinical Trials Registry India: CTRI/2018/04/013001 retrospectively registered on April 4, 2018.

## Background

Chronic, non-communicable diseases have replaced infectious diseases as the number one cause of mortality and disability globally [1–6], and mental disorders are among the leading causes of disability worldwide [7]. In India, the prevalence of common mental disorders (CMD) including depressive and anxiety disorders has been estimated to affect 30-34% of primary care patients [3, 8]. The majority of patients with CMD visiting primary health care centers (PHCs) present with multiple somatic symptoms and are often misdiagnosed, resulting in the receipt of ineffective, symptomatic treatments [9, 10]. In a survey of 12,886 patients visiting a clinic in South India who were participating in a community mental health program it was observed that major depressive disorder and dysthymia accounted for 34% and 22%, respectively, of the total burden of mental illness [11]. Although depression can be effectively treated in PHCs in approximately 60-80% cases, only 10-25% of these cases seek treatment [11], typically due to lack of awareness or perceived stigma and discrimination [11, 12].

Mental disorders increase the risk for both communicable and non-communicable diseases and many of these conditions in turn increase the risk for CMDs [13–15]. Depression, independent of other risk factors in an otherwise healthy person, increases the risk of developing cardiovascular disease (CVD) and adversely impacts cardiac outcomes [16–18]. The depression-CVD co-morbidity not only results in increased mortality but also greater morbidity and disability [19]. Mental disorders and CVD constitute nearly a fifth of the disease burden in India in terms of disability adjusted life years lost [20]. Major coronary risk factors, such as high blood pressure, dyslipidemia, insulin resistance and diabetes are also escalating in this population and correlate positively with the increase in coronary disease [21]. In a study of 103 patients with a recent myocardial infraction attending a cardiology outpatient tertiary care center in Northern India, 25.2% of patients were diagnosed with anxiety or depressive disorder on the Mini International Neuropsychiatric Interview (MINI) [22]. Joseph and Srinivasan [23] reported that 23% of patients who presented with chest pain to a tertiary care facility had diagnosable coronary artery disease (CAD), and the psychological distress in CAD was due to co-morbid psychiatric conditions.

Most chronic non-communicable diseases share modifiable behavioral risk factors, including excessive fat and salt intake, sedentary behaviors, and harmful use of alcohol and tobacco consumption [3, 24], making them excellent candidates for integrated intervention programs [25]. The US-based TEAM care study found that integrating depression and chronic disease care among patients with diabetes mellitus (DM) and/or CAD resulted in greater overall 12-month improvement in glycosylated hemoglobin, LDL cholesterol, systolic blood pressure, depressive symptoms, and quality of life [26, 27]. These interventions not only improve outcomes but are cost-effective too [27, 28]. Both the COPES and SUPRIM trials have found that treatment for depression among patients with CAD was associated with a lower risk of secondary cardiovascular events [29, 30]. The rationale behind integration of the management of CMD with CVD and DM is thus four-fold: 1) both are chronic conditions requiring multiple encounters with the health system and adherence to extended duration treatment regimens; 2) there are considerable cross-benefits of the behavioral intervention strategies; 3) integration of mental health services with those provided for CVD and diabetes are cost effective and could contribute to strengthening health systems by providing shared resources [27, 31, 32]; and 4) barriers to effective treatment are similar, including over-medicalization of diagnosis and management, lack of basic screening and diagnostic tools, insufficient affordable financing mechanisms and lack of trained health-care providers [33].

Many resource-constrained setting, including India, face a shortage of physicians and nurses [34]. India has less than one psychiatrist for every 300,000 population [35], however, in rural areas, which account for 70% of India’s population, this ratio has been estimated at less than one per million [35, 36]. The availability of other mental health professionals such as psychologists, social workers and psychiatric nurses is even less, pointing to the need to train PHC providers and community health workers in identifying and treating these disorders [37–39]. In response, community lay health workers have been successfully trained to improve a range of physical and mental health outcomes. The content of such programs has ranged from cancer education and screening [40] and asthma management [41] to women’s reproductive health [42]. Shifting to lower-level providers and caregivers for on-going patient support shows promise for achieving better outcomes at lower cost [4, 43, 44]. Though India has a long history of use of community health workers in ‘task-shifting’, this has occurred primarily in the fields of maternal and child health and tuberculosis and less so in the field of chronic diseases. A recent study found that non-physician health workers and ‘expert physicians’ agreed on how to correctly apply the World Health Organization (WHO) Cardiovascular Risk Management Package 80% of the time across PHCs in India and Pakistan [45]. In rural Andhra Pradesh, India, non-physician health workers have been found effective in identifying adults with high cardiovascular risk, by following a simple algorithm [43].

The collaborative care model [46–52] involving case managers and consulting psychiatrists in support of primary care providers in the treatment of mental disorders has been successful in providing integrated care for mental health and medical illness in PHC settings [13, 47] and was more effective than standard care (50% vs. 19% reduction in depressive symptoms) in U.S clinics among patients aged 60 and older with major depression and/or dysthymic disorder [47]. A meta-analysis of 37 randomized control trials (RCTs) and 12,355 patients showed that both short term and long term outcomes for depression improved significantly for patients in the collaborative care arm [53]. The integrated collaborative care model has also been found to be cost-effective targeting both depression [54] and chronic medical illness, including diabetes [27, 28, 55]. It has been adapted for primary care with different racial and ethnic groups [56], among patients with different co-morbid conditions, including depression and cancer [57], and depression and DM [58]. The collaborative care model has also demonstrated sustainability in PHC settings [26, 59]. While the collaborative care model has been primarily tested in Western settings, a stepped collaborative care model has been used previously [60] in Indian PHCs to treat mental disorders. Our study extends and adapts the integrated collaborative care model for patients diagnosed with depression and chronic medical conditions in limited resource settings.

The protocol described in this article is an ongoing randomized controlled-trial to implement and evaluate a multi-level community based collaborative care model in rural Ramanagaram district in the state of Karnataka, to improve screening, diagnosis, and treatment of depression in India among patients with co-morbid CMD and either DM or CVD at PHC. The aims of our study are; 1) To examine if community-based screenings for depression, anxiety, DM and CVD risk factors during community health fairs (a) increase subsequent diagnosis of these disorders in PHCs; and/or (b) lead to better linkage and retention in care as compared to the standard PHC based screening. We are using accredited social health activists (ASHAs) to raise awareness and provide outreach for the community health fairs; 2) Implement and evaluate the effects of providing staff training to PHC staff in the collaborative care model of integrated mental health (depression and anxiety) and chronic disease (hypertension, DM, and CVD) as compared to the enhanced standard care model. In addition, we are implementing a community-based risk factor reduction groups (Healthy Living Intervention), co-facilitated by ASHAs, to target risk factors common to both mental illness and chronic physical disease, with group session topics in exercise, diet, adherence to medical regimens, ocial support, coping skills, and problem solving skills; 3) evaluate the effects of the clinic and community-based intervention for co-morbid primary care patients compared to the enhanced standard treatment services.

## Methods

### Study design and overview

**Fig. 1:**
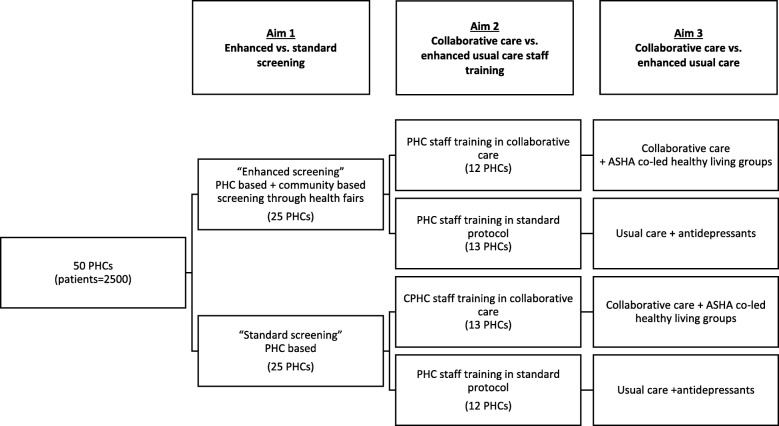
Study design flowchart

As shown in Fig. 1, the study was designed to implement and evaluate the effects of a collaborative care intervention on the screening, diagnosis and treatment of depression among rural Indians with depression or anxiety and either hypertension, diabetes or CVD, who live in villages associated with 50 PHCs in rural Karnataka. The design of randomized controlled trial is described below and compares 1) the enhanced health fair screening condition to standard PHC screening; and 2) community-based and collaborative care to enhanced standard treatment.

Ethics approval was obtained from the Institutional Ethical Review Board at St. John’s Medical College and Hospital and Committee on Human Research, University of California, San Francisco. All adverse events are identified by trained field staff and assessed by the Principal Investigators. Participants with adverse events are referred to the medical officer at the PHC and or at district hospital for immediate medical intervention as appropriate. Deaths are reported to the St. John’s Institutional Ethics Committee, UCSF IRB, and the Data Safety and Monitoring Board within 5-10 days per protocols approved by the IRBs, the funder and the government of India. Possibly study-related non-serious adverse events are reported to the respective ethics review board by the principal investigators, according to protocol. This study is monitored by a Data Safety and Monitoring Board which consists of a psychiatrist, an expert in community medicine/rural health and a statistician with experience conducting RCTs. The board reviews all procedures biannually related to the protection of study participants, including confidentiality procedures and reports of adverse events. The board has access to data to determine if the trial can continue or needs to be terminated.

### Conceptual framework

We used a multi-level framework based on previous literature on collaborative care to improve screening diagnosis and treatment of depression in primary health settings, and our own extensive work in community medicine and behavior change in India to guide our adaption of the primarily western collaborative care model. Our intervention offers several key innovations for treatment of co-morbid patients by identifying and targeting common risk factors with the help of ASHA and by training PHC staff in the collaborative care model. The “healthy living” intervention described below uses a novel package of evidence-based strategies in a group setting. Our multi-level intervention represents a timely, novel, sustainable and comprehensive approach to co-morbid diagnosis and disease management by integrating multiple existing health care staff and structures into a continuum of care through community based screenings, linkage to and retention in care and ongoing support through the use of community-based groups and mobile technology.

We incorporated constructs from the *Social Ecological Model* [61–66] and *Social Cognitive Theory* [67, 68] because of its emphasis on both interpersonal interactions as well as on specific strategies that promote behavior change; all important to reducing symptoms of depression, anxiety and stress and to increasing health promoting behaviors. In particular, the following three features of this theory guide the delivery of our intervention 1) people and their environments interact continuously, and behaviors are the result of this dynamic bi-directional interaction. Our intervention facilitates this interaction through ASHA at the community level through health fairs, PHC staff at the clinic level, and social support through healthy living groups; 2) By including group-based activities, participants can learn from and motivate each other; 3) The intervention emphasizes skills building, while assisting participants to break down challenges into manageable components necessary to enable sustainable behaviors.

### Setting and randomization

This study is conducted in collaboration with 50 PHCs in rural Ramanagara district of Karnataka state in southern India, each PHC serves a population of 30,000. A typical PHC has a medical officer, pharmacist, staff nurses, female multipurpose health workers (auxiliary nurse-midwives), male multipurpose health workers, ASHA workers, a laboratory technician/assistant, driver and helpers. It includes both outpatient and inpatient areas with four to six beds, as well as space for counseling, minor emergencies, a labor room, a pharmacy and a laboratory. The PHCs were assigned an identification number, and 25 PHCs were randomly assigned by the study statistician using a pseudo-random number generator to the enhanced screening arm linked to community health fairs and the remaining 25 were assigned to the standard screening arm. Subsequently, half of the enhanced screening and standard screening PHCs were randomly assigned to the collaborative care arm and the remaining half was assigned to the standard care treatment condition (Fig. 1). Participants randomized to the intervention group will be stratified by gender. While participants and intervention staff are not blinded, assessment staff are blinded to group assignment.

### Enhanced screening vs standard screening conditions

The “enhanced” screening condition is implemented in 25 of our 50 PHC catchment areas, allowing us to examine whether the added community-based screening through community health fairs a) increases identification of patients with co-morbid diagnosis (Table 1); and b) improves linkage to and retention in care, compared to co-morbid patients identified via standard PHC-based screening. Specific recruitment procedures for each setting are described below, followed by common screening procedures and tests.

**Table 1:** Initial and Confirmatory Screening Criteria

Screening stage	Inclusion criteria at each stage
Initial Screening Inclusion Criteria *Enhanced screening (health fair or PHC)* *Standard screening (PHC only)*	*Age:* 30 years or older *Mental competency:* Modified Short Blessed Cognitive Test [103] score ≤7 *General psychological distress*: The Kessler-10 [81] score ≥6 *One or more of the following cut-off measures for diabetes, hypertension, and/or CVD:* • Capillary Blood Sugar ≥160 mg/dL • Blood pressure ≥ 140/90 mmHg • Possible Angina: Rose angina questionnaire [104]^a^ • Self-reported physician-diagnosed history of DM, hypertension or ischemic heart disease
Confirmatory Screening Inclusion Criteria (PHC only)	*Depression/anxiety*: The Mini-International Neuropsychiatric Interview (MINI) [82] *One or more of the following clinical measures for diabetes, hypertension, and/or CVD:* • Blood pressure ≥ 140/90 mmHg • Capillary blood sugar ≥160 mg/dL • Physician-diagnosed history of DM, hypertension or ischemic heart disease (patient must show prescription or medication) • Possible rose angina^b^
Exclusion Criteria	• not mentally competent to provide consent per the standard screener [103], answer to study measures and/or participate in the intervention. • Participants who do not provide contact information

^a^ Patients who screen negative for hypertension and DM, but have possible angina on the rose angina questionnaire will be invited to the confirmatory screening

^b^ Patients who screen positive on the rose angina measure at initial screening and positive on the MINI during the confirmatory screening, but negative on hypertension and DM are marked as tentatively enrolled on the confirmatory screening form. At the baseline assessment electrocardiogram and cholesterol to check for CVD will confirm eligibility

At the enhanced screening PHCs, nurses and ASHA run health fairs in villages that are a part of our collaborating PHC catchment areas. Prior to conducting the fairs, ASHAs raise awareness and provide outreach of the upcoming health fair through announcements at community events and meetings, poster, brochures, and door-to-door visits to community members. One health fair is held per week during the five-week recruitment period. The health fair provides an opportunity for people in the community to receive a free health check-up.

Screening occurs in two phases, during the initial screening and confirmatory screening (Table 1). The initial screening is held at the health fair or at the associated PHCs in the enhanced screening condition and at the PHC only for the standard screening condition. Interested patients give written informed consent to study staff for the screenings, and those meeting the eligibility criteria during the initial screening are invited to a confirmatory screening at the PHC. Patients who meet the eligibility criteria during the confirmatory screening are invited to participate in the study. Eligible participants receive information about the study verbally as well as in written form if literate, including details about the intervention, study protocol, randomization process, time commitment and potential risks and benefits. Participants are informed that participation is voluntary, there are no negative consequences for refusing to participate, and that consent can be withdrawn at any time during the study without any repercussions. Participants receive a copy of the study information sheet and informed consent. Illiterate participants have an option of providing verbal consent or a thumb print. In those cases, a witness, unaffiliated with the study, also signs the consent form.

#### Inclusion and exclusion criteria

Participants, who are 30 or older, with co-morbid CMD (Depression or Anxiety Disorder) and either hypertension, diabetes, or ischemic heart disease, and who are willing and able to consent and be followed for 12 months are considered eligible for inclusion in the study.

### Collaborative care intervention design

The proposed multi-level “Healthy Living” intervention has been designed to promote long-term mental and physical health among the participants, by training PHC staff and ASHAs in the collaborative, stepped care model and by providing patients with skills that can be incorporated into their lifestyles. The stepped care model includes referrals of suicidal patients to the district psychiatrist and additional referrals for abnormal lab values, and the support of our psychiatry consultants during their weekly calls. The content and format are guided by our conceptual model [64, 66, 69, 70] and include strategies that can be easily integrated into existing health care structures and that have been found effective in previous studies [27, 28, 32, 39, 55, 59, 60, 69–77].

#### Collaborative care staff training

Staff in the 25 intervention PHCs receive training sessions in the collaborative care model [26] by psychiatrists from St John’s Medical College, who have volunteered to take on the role of “consultant psychiatrist” [78]. The PHC staff training is designed to enable them to effectively integrate treatment of CMDs into their regular practice in patients with co-morbid medical conditions. A modified version of the IMPACT model [48, 78] was adapted for our setting to maximize the likelihood of sustainability. PHC staffs are required to undergo one full day of interactive group training. The morning session trains all PHC staffs on management of chronic non-communicable diseases at the clinic level and the second half focuses on collaborative care in mental health. Primary care physicians are trained to identify and treat patients presenting with a CMD. The PHC nurses are trained to function as “care managers,” and help with tracking patient progress. Support is also being provided by the consulting psychiatrists, who provide both routine caseload, diagnostic, and treatment consultation for difficult cases, which may include referral recommendations when additional care is needed. Other care team members include the PHC pharmacist, trained to educate patients and their caregivers about their medication regimen, side effects and adherence. Finally, the ASHAs are trained in risk factor screening and modification, and act as a liaison between the PHC, patients, families and community. They co-facilitate the healthy living groups and provide appointment reminders through home visits.

#### Clinic-based intervention

Participants in the 25 intervention PHCs, receive diagnostic test and clinical treatment for both their mental illness and chronic disease by the PHC care team trained in comprehensive integrated mental health and CVD care using the stepped collaborative care model described above [39, 74].

#### Community-based intervention

Participants in the intervention PHCs are given an appointment to participate in a 12-month, healthy living group, designed to target risk factors important in management of depression, anxiety, DM, and CVD (Table 2). Each group includes eight to 10 same-sex participants and held in an easily accessible venue in the community. The first 12 weekly sessions are facilitated by a master’s level counselor and co-facilitated by an ASHA, who subsequently provides nine monthly sessions focused on behavior maintenance. The behavioral change strategies used are based on principles of social cognitive theory, such as observational learning, setting manageable goals, practice and getting feedback, building self-efficacy and skills training [69, 70].

**Table 2:** Community-based Healthy Living Group Session Topics and Exercises

Session #	Topics covered	Exercises
1	CVD risk factors and diabetes	Discuss modifiable and non-modifiable behavioral risks
2	Psychological well-being, depression, anxiety and stress	Identify sources of and strategies to reduce depression, anxiety, and stress
3	Role of nutrition in disease	Review eating habits and develop plan to eat healthier
4	Improving physical and emotional health with exercise	Strategies for overcoming obstacles and creating a habit to exercise on a regular basis
5	Smoking and smokeless tobacco cessation	Discuss harmful effects of tobacco, triggers, and strategies for quitting
6	Alcohol use	Discuss harmful effects of alcohol, triggers, and strategies for quitting
7	Social support for behavior change	Sources of emotional, practical, and informational, strategies for approaching family, friends, and community for support, identify social support needs. Communication skills to use with unsupportive people.
8	Quality of life	Assessing quality of life and identifying strategies to improve quality of life
9	Tools for initial behavior change	Self-monitoring, goal setting, self-reinforcement, setting up an environment that supports change
10	Review of sessions 1-9	Identify successes and barriers to behavior change
11, 12	Long term maintenance of behaviors. Different strategies needed for initial change	Setting and reaching behavior change goals by using strategies learned in previous sessions
Monthly	Behavioral maintenance session	Review accomplishments, problem solving barriers, revising plans, utilizing group support

#### Session format

Each begins with breathing exercises for relaxation known to be effective in both CMD and CVD [79, 80]. The interrelationship between thoughts, emotions, behaviors and their impact of health are discussed to set the stage for the introduction of cognitive techniques. A list of common stressful life situations are developed by the group and used as examples for subsequent problem-solving skills training and coping skills training. Participants are encouraged to set both short term and longer-term goals and to make a commitment to change at the end of every session and reviewed and reinforced in the following-section. In addition, participants are also encouraged to set up buddy systems within the group and establish informal peer support networks.

We anticipate that our integrated intervention will have both direct and indirect beneficial effects on the families and communities associated with the Intervention PHCs. ASHAs involved with the integrated intervention groups meet with every participant’s family during bi-monthly home visits and encourage them to support the participant’s new healthy lifestyle. Participants themselves are encouraged to act as dissemination agents by sharing the knowledge and skills learned during the Healthy Living groups with their families.

Implementation and adherence to intervention protocols are documented and monitored through weekly reports of HLG sessions, weekly psychiatry consultation calls between PHC medical officers and the consulting psychiatrist, and through observation of the intervention sessions by an independent monitor who completes a check-list to ensure that all components are covered. In addition, the intervention coordinator makes weekly visits to the PHCs to ensure that all participants are appropriately referred for care. All intervention staff are trained and certified in all components of the intervention.

### “Enhanced” standard treatment

All staff in the PHCs that have been randomized to Enhanced Standard Treatment will receive a full day of basic training in established clinical protocols set by the state of Karnataka. For ethical reasons, since standard PHC treatment often includes inappropriate use of vitamins and anxiolytics, a psychiatrist leads the afternoon session training on how to treat CMDs per standard treatment protocols.

Patients in the standard treatment arm will receive usual care per the standardized protocols developed by the State. We will also ensure that any patient who is diagnosed as moderately to severely depressed has access to effective anti-depressant medication by referring eligible patients to a psychiatrist located in the nearest district hospital. Patients identified as at high risk for suicide are also referred to district hospital psychiatrists at screening and assessments per study protocol. In addition, any abnormal clinical results (e.g. hypertension, DM, etc.) found at screening and cohort assessments receive an appropriate referral.

### Outcome measures and schedule

The vast majority of the study measures have been used previously in India. Remaining measures (internalized stigma of mental illness, patient satisfaction and clinical vignettes), were adapted and pilot tested during our start-up phase to ensure that they are appropriate for our specific study population and setting. All measures have been translated into Kannada and back translated.

All cohort participants are assessed at baseline, 6 weeks, 3 months, 6 months and 12 months. To minimize attrition, we collect extensive contact information from all participants at enrollment. This includes mobile phone numbers, as well as street addresses, information about landmarks, and the name and phone number of someone who always knows how to reach them. This information is verified and updated during each assessment visit, HLG sessions visit and tracking phone calls and visits. All research materials will be coded with ID numbers only and linked to contact information on a separately stored document kept under lock and key.

To examine if community-based health fair screenings increases subsequent diagnoses in the PHC of patients with co-morbid mental health and chronic disease diagnoses, we use the Kessler-10 [81], MINI [82], and the clinical measures for diabetes and CVD outlined in Table 1. To screen for psychological distress at the initial screening using the Kessler-10, a brief standardized questionnaire that correlates with other commonly used depression screening questionnaires and with the DSM IV diagnoses of both depression and anxiety disorders [81, 83]. At the subsequent confirmatory screening, MINI is used to confirm the diagnosis of anxiety or depressive disorder as per DSM-IV guidelines. We also assess suicidal ideation based on items from the MINI and refer participants at high suicidal risk to the district psychiatrist for further management and treatment.

Linkage and retention in care is measured by the proportion of participants who started treatment and the proportion of these participants who were retained in care throughout the study.

To evaluate the effects of the clinic and community-based intervention for co-morbid primary care patients compared to the enhanced standard treatment services we measure severity of anxiety and depressive symptoms, blood pressure, body mass index and measures for diabetes and cardiac conditions (Table 3).

**Table 3:** Outcome measures and study schedule

Measure	Screening	Baseline	6 weeks	3 months	6 months	12 months
Clinical Measures						
Systolic blood pressure	X	X	X	X	X	X
Diastolic blood pressure	X	X	X	X	X	X
Blood sugar	X					
BMI (weight/height)	X	X				X
Weight		X	X	X	X	X
Waist circumference		X	X	X	X	X
Electrocardiogram		X				X
Total cholesterol (with lipid profile)		X		X	X	X
LDL (Lipid profile)		X		X	X	X
HDL (Lipid profile)		X		X	X	X
Triglycerides (Lipid profile)		X		X	X	X
Glycosylated hemoglobin		X		X	X	X
Serum creatinine		X		X	X	X
Urine creatinine		X				X
Urine microalbumin		X				X
Questionnaires						
Rose angina questionnaire [104]	X					
The Kessler-10 [81]	X					
Modified Short Blessed Cognitive Test [103]	X					
MINI [82]	X					
PHQ-9 [85]		X	X	X	X	X
GAD-7 [84]		X	X	X	X	X
Patient perception					X	X
Fagerstrom Test for Nicotine Dependence (FTND) and FTND-Smokeless Tobacco [105, 106]		X	X	X	X	X
Alcohol Use Disorders Identification Test (AUDIT) [107]		X	X	X	X	X
Food Frequency [108]		X				X
International Physical Activity (IPAQ) [109]		X		X	X	X
Medication adherence (VAS) [110]		X	X	X	X	X
Diabetes self-management questionnaire [111]		X	X	X	X	X
World Health Organization (WHO) Disability Assessment Schedule 2.0		X				X
Demographics		X	X	X	X	X
Social support [112]		X		X	X	X
WHO Quality of life (WHOQOL-BREF) [113]		X			X	X
Integrated Stigma for Mental Illness Scale (ISMI) [114]		X		X	X	X

#### Severity of anxiety and depressive symptoms

The Generalized Anxiety Disorder Scale (GAD-7) [84] and the Patient Health Questionnaire Depression Scale (PHQ-9) is used to assess severity of anxiety and depression, respectively [85].

#### Blood pressure (BP)

Systolic and diastolic blood pressure is measured using a standardized protocol. Two measurements are taken and the average of the two readings is calculated [86, 87]. Hypertension is defined as elevated blood pressure (average systolic BP (SBP) ≥ 140 mmHg and/or an average diastolic BP (DBP) ≥90 mmHg) or higher levels of hypertension if SBP ≥ 160 mmHg and/or an DBP ≥ 95 mmHg.

*Body Mass* Index (BMI) is calculated as weight (kg) divided by height (m^2^) [78]. Waist circumference is measured in centimeters.

#### Measures of diabetes and cardiac risk

Lipids (total cholesterol, LDL, HDL and triglycerides), *kidney function* (serum creatinine, urine creatinine and urine microalbumin) and glycemic control (HbA1c) are measured using standard assays. Dyslipidemia are defined as: LDL > 130 or HDL < 40 mg/dL [88]. We selected HbA1c because it is not affected by short-term dietary changes and strongly correlates with disease severity [89].

### Collaborative care PHC staff training outcome measures

Knowledge and Skills related to collaborative care are assessed using clinical vignettes followed by a set of questions on CMD and CVD screening, treatment, case management, communication, & use of consultation and referrals, tailored to each type of health professional at the PMC. Vignettes are effective in evaluating the benefits of training [90–93] including mental health training among primary care physicians in India [94].

#### Patient perceptions

To minimize socially desirable responses [95], we developed and administer a behaviorally anchored measure that targets patient perceptions of staff in terms of 1) patient interactions; 2) providing relevant information; 3) active listening and answering questions; and 4) addressing risk behaviors.

All data collected in interviews and laboratory tests are de-identified and uploaded to an encrypted password protected database. Double data entry occurs within 3 days of data collection. The data are monitored on an ongoing basis for completeness and accuracy.

### Statistical analyses

Preliminary analyses will include examination of the reliability of scales (e.g. PHQ-9, GAD-7), attrition analyses to compare respondents with complete data to those who do not complete the study with regard to group assignment and baseline demographics, and comparisons between the two intervention arms, to check for group balance. We will use chi-square tests for unordered categorical variables, Kruskal Wallis tests for ordinal or non-normal continuous data, and ANOVA for normal continuous variables.

#### Screening

To test the hypothesis that, compared to standard screening, enhanced screening will result, on an average, in more people being identified as co-morbid for CMD and CVD/DM during the confirmatory testing at the PHC, we will use a Poisson regression model - or in case of over dispersion, a negative binomial model - with PHC as the unit of analysis, and population size in the catchment area of the PHC included as an offset.

Secondary analysis will explore subsequent linkage and retention in care of enrolled participants first screened at the community health fairs compared to those first screened at PHC. These participants will be compared by examining the proportion of participants who started treatment both by the 6 week follow-up, as well as the proportion retained throughout the study. We will use a mixed-effects logistic regression model with a random intercept for PHC.

#### Intervention

The primary, intention-to-treat (ITT) analyses will evaluate the impact of the intervention, both in terms of a) return to below-threshold levels for CMD and CVD/DM risk (i.e. dichotomized outcomes), and b) improvement in the levels of the continuous CMD and CVD/DM measures (depression and anxiety scores, BP, HbA1c, LDL, and serum creatinine levels) via logistic and linear mixed-effects regression models respectively, with repeated measures nested within individuals, and individuals nested within PHC, and random intercepts for individuals and PHC [96, 97]. The trajectories of the continuous variables will be examined via the time-by-intervention interaction effect. We will run separate regressions for the various outcomes, to allow us to detect if different variables react to the intervention at different rates. To prevent Type I error inflation, we will lower α accordingly. In all models covariates will be included as necessary.

The sample size of 1250 in each intervention arm (50 participants per PHC), was determined based on achieving 80% power for the ITT analyses regarding the effect of the intervention, and was calculated as follows: we assumed an attrition rate of 20%, and an intra-class correlation (ICC) of 0.1 to account for clustering of participants in PHCs, which reduces the initial sample size to an effective sample size of *n* = 204/group. Pooling data across the three post-intervention measurements triples this number, and subsequent adjustment for repeated measures with an assumed ICC = 0.5, results in a final *effective* sample size of *n* = 306 person-time observations per group. Based on previous research, we assumed 40% of control group participants to recover [98, 99]. With α = .025, the minimum detectable effect size at 80% power is 12% more of the intervention group participants recovering [100]. This is a small effect size according to Cohen [101], and comparable to earlier studies in the US [26]. To test the screening hypothesis, with 25 PHCs per screening condition, 80% power and α = 0.05, the minimum detectable effect in a Poisson regression is 2.1 times as many co-morbid cases identified with the enhanced compared to the standard screening [102]. Although this is a large effect, we deemed it attainable, given the documented under-reporting of mental health in standard care [10, 11], and the intensive nature of our enhanced screening.

#### Current status of the study

Intervention and assessments are ongoing as of August 3, 2018. During the final year of our research, a dissemination meeting will be held for key stakeholders including local, state, and national government officials, and hospital administration officials.

## Discussion

This randomized controlled trial evaluates the effectiveness of multi-level integrated clinic and community-based intervention model for common mental disorders co-morbid with diabetes and cardiovascular conditions compared to enhanced care as usual. In addition, we test the effectiveness of using ASHAs as link workers between the community and the PHC in increasing referrals of such patients to the clinic and improving retention rate in the treatment regimen. A novel feature of this proposal includes group sessions that target risk behaviors common to both depression and co-morbid medical conditions. Our intervention builds on collaborative care model and is dependent on trained PHC physicians to deliver evidence based intervention for both CMD and co-morbid medical conditions and directly addresses the scarcity of trained mental health professionals in rural India. It has a high potential for scale up and sustainability as it builds on strengthening the linkages between the community and the existing Government programs.

## Abbreviations

ASHA: Accredited social health activist
BMI: Body mass index
BP: Blood pressure
CAD: Coronary artery disease
CMD: Common mental disorder
CVD: Cardiovascular disease
DM: Diabetes mellitus
GAD: Generalized anxiety disorder
ITT: Intention to treat
MINI: Mini international neuropsychiatric interview
PHC: Primary health center
PHQ: Patient health questionnaire
RCT: Randomized controlled trial
WHO: World Health Organization
